# Preparation and Antimicrobial Activity of a Film-Forming Polyhexamethylene Biguanide Teat Disinfectant

**DOI:** 10.3390/ijms242417444

**Published:** 2023-12-13

**Authors:** Yixing Lu, Di Wang, Yongxiang Zhang, Yueying Hu, Jiaxuan Lu, Zhenling Zeng, Dongping Zeng

**Affiliations:** 1 Guangdong Provincial Key Laboratory of Veterinary Pharmaceutics Development and Safety Evaluation, College of Veterinary Medicine, South China Agricultural University, Guangzhou 510642, China; lyx2021@stu.scau.edu.cn (Y.L.); 20212027048@stu.scau.edu.cn (D.W.); yongxiang@stu.scau.edu.cn (Y.Z.); huyueying02@stu.scau.edu.cn (Y.H.); jiaxuan-lu@stu.scau.edu.cn (J.L.); 2 National Risk Assessment Laboratory for Antimicrobial Resistance of Animal Original Bacteria, Guangzhou 510642, China

**Keywords:** mastitis, disinfectants, polyhexamethylene biguanide, antibacterial activity, prevention

## Abstract

Bovine mastitis caused by infectious pathogens can lead to a decline in production performance and an increase in elimination rate, resulting in huge losses to the dairy industry. This study aims to prepare a novel dairy cow teat disinfectant with polyhexamethylene biguanide (PHMB) as the main bactericidal component and to evaluate its bactericidal activity in vitro and its disinfection effect in dairy cow teats. PHMB disinfectant with a concentration of 3 g/L was prepared with PVA-1788, propylene glycol and glycerol as excipients. When the dilution ratio is 1:4800 and the action time is 5 min, the PHMB teat disinfectant can reduce the four types of bacteria (*S. agalactiae* ATCC 12386, *S. dysgalactiae* ATCC 35666, *S. aureus* ATCC 6538, and *E. coli* ATCC 8099) by 99.99%. PHMB teat disinfectant applied on the skin of rabbits with four bacteria types achieved an average log_10_ reduction greater than 4. After 30 s of PHMB teat disinfectant dipping, the bacteria of cow teats were counted prior to disinfection. The mean log_10_ reduction in bacteria on the skin surface of 12 cows ranged from 0.99 to 3.52 after applying the PHMB teat disinfectant for 10 min. After 12 h, the PHMB teat disinfectant achieved an average log_10_ reduction in bacteria from 0.27 to 0.68 (compared with that prior to disinfection). These results suggested that PHMB teat disinfection has the potential to prevent and treat mastitis-causing bacteria in dairy herds.

## 1. Introduction

Mastitis caused by infectious pathogens remains a devastating disease concerning dairy cattle, affecting animal welfare and causing huge economic losses to the dairy industry through reduced performance and increased culling rates [1]. Bovine mastitis is one of the major problems facing the dairy industry and is usually caused by a bacterial infection [2,3]. The teat orifice is the first line of defense in preventing mastitis pathogens from entering the udder. Teat disinfection during pre- and post-milking has been shown to reduce the bacterial count on the teat skin, making it an important tool in reducing intramammary infections and mastitis [4]. Topical formulations containing antimicrobial agents ensure high concentrations are delivered directly to the site of infection, reducing bacterial resistance, minimizing unwanted host responses and reducing the risk of environmental contamination [5]. Guanidine compounds have been found to exhibit diverse biological and pharmacological activities [6]. Notably, guanidine groups are present in various antimicrobial agents, including streptomycin and trimethoprim [7]. Moreover, Guanidine disinfectants, such as chlorhexidine, have been employed for teat disinfection in dairy cows [8,9].

**Figure 1:**
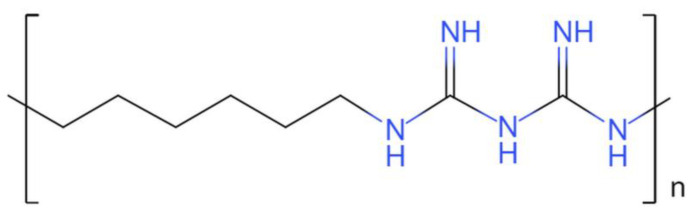
Chemical structure of PHMB.

Polyhexamethylene biguanide (PHMB) is a cationic polymer with a broad antimicrobial spectrum, well tolerated and low risk [10]. The chemical structure of PHMB is shown in Figure 1. The guanidine group on PHMB can bind to the phosphate head group of the bacterial cell membrane, resulting in the outflow of cell contents and cell death [11]. PHMB has been successfully utilized in ophthalmology and dentistry [12,13]. A study has demonstrated the therapeutic efficacy and tolerability of a vaginal solution containing PHMB for the treatment of bacterial vaginosis [14]. Bacterial cellulose wound dressings incorporating PHMB offer potent benefits for would treatment, including wound protection, acceleration of wound healing, pain reduction, and antimicrobial effects [15]. A cohort study has shown that a concentration of 0.04% PHMB was effective in preventing infections in traumatic soft tissue wounds, outperforming 1% povidone–iodine, 4% hydrogen peroxide and undiluted Ringer’s solution [16]. Llorens et al. have reported the development of polylactide nanofibers loaded with PHMB, which create a 3D biodegradable scaffold with antibacterial properties. Polylactide scaffolds loaded with PHMB exhibit antibacterial properties that hinder the adhesion growth of bacteria while also being biocompatible with fibroblast and epithelial cell adhesion and proliferation [17]. Peng et al. discovered that PHMB coating on titanium alloy surfaces was densely packed with block polymer, which did not compromise the cytocompatibility of the substrates but effectively inhibited bacterial growth, thereby reducing bacterial-associated infections in rats [18]. These findings demonstrate the promising potential of PHMB in the medical field due to its stability and low cytotoxicity [19,20].

PHMB has been extensively utilized in the advancement of biomaterials; nonetheless, there is a paucity of research regarding its application as a bovine teat disinfectant. Furthermore, the implementation of film-forming medicated bath liquid has demonstrated the ability to generate a delicate protective film on the skin [21]. This not only facilitates the disinfection of the teat and its surrounding skin but also addresses the issue of dry and cracked teats resulting from the limited moisturizing duration of conventional medicated bath liquid. In this study, a film-forming teat disinfectant with PHMB as the main bactericidal component was prepared and its disinfection effect was evaluated. We used four indicator bacteria (*S. agalactiae* ATCC 12386, *S. dysgalactiae* ATCC 35666, *S. aureus* ATCC 6538, and *E. coli* ATCC 8099) to determine the in vitro antibacterial activity and simulated skin disinfection of PHMB teat disinfectant. In addition, the effect of PHMB teat disinfectant on killing bacteria in bovine teat skin was evaluated. 

## 2. Results

### 2.1. Preparation and Optimization of PHMB Teat Disinfectant

Four different film-forming agents, PVA-1788, sodium carboxymethyl cellulose, chitosan and povidone-K30, were selected for preliminary screening. The results showed that the sodium carboxymethyl cellulose test group produced a white flocculent precipitate, and the chitosan test group was insoluble in water and crystalline. Povidone-k30 and PVA-1788 can be dissolved in water with PHMB in a short time, and the liquid is transparent and clear. Furthermore, 1–10% of PVA-1788 and 1–15% of povidone-K30 were screened. When the concentration of PVA-1788 ranges from 5% to 7%, the solution state is mucus, with good film formation and good skin adhesion. The optimum concentration ratios of the film-forming material (PVA-1788), humectant (propylene glycol) and antifreeze (glycerol) were determined via an orthogonal test. The PHMB teat disinfectant was prepared under various conditions, such as the volume of PVA-1788 from 5% to 7%, different volumes of propylene glycol from 20% to 30%, and various volumes of glycerol from 5% to 15%. The film formation time and the appearance properties of the solution were combined for scoring. The PHMB disinfectant with the highest score was obtained under the following optimal conditions: 6% of PVA-1788, 30% of propylene glycol, and 5% of glycerol. Six batches of PHMB teat disinfectant were prepared according to the optimal conditions, and the average content was 2.83 g/L (Table 1). After 90 days of storage at 37 °C, the average content was 2.76 g/L. The result shows that the prepared disinfectant is relatively stable.

### 2.2. In Vitro Antibacterial Activity

The mean reduction rate following suspension with PHMB teat disinfectant under the various test conditions (exposure times and dilutions) for *E. coli* ATCC 8099, *S. aureus* ATCC 6538, *S. agalactiae* ATCC 12386, and *S. dysgalactiae* ATCC 35666 are shown in Table 2 and Table 3. The bactericidal effect of the PHMB disinfectant against *S. agalactiae* and *S. dysgalactiae* was superior to that of *E. coli* and *S. aureus*. When the dilution ratio is 1:4800 and the action time is 5 min, the PHMB disinfectant can reduce the four bacteria types by 99.99%. For the four indicator bacteria, the disinfectant activity of PHMB continued to decrease across the dilution range and then increased with increasing contact time.

### 2.3. Factors Influencing Disinfection Effect

The bactericidal activity of the PHMB teat disinfectant should be significantly independent of organic matter, temperature and pH. The germicidal efficacy of disinfectants in the presence of organic matter, different temperatures and different pH values was tested. When the proportion of organic matter was 5%, 10%, 15% and 20%, the killing rate of PHMB teat disinfectant on the four indicator bacteria reached more than 99.99% in the action time of 5 min to 15 min. At the temperatures of −20 °C, −10 °C, 0 °C, 10 °C, 20 °C, 30 °C and 40 °C, the killing rate of PHMB disinfectant on the four indicator bacteria reached 99.98% within 5 min. When the pH was 4–10, the killing rate of the four types of indicator bacteria reached 99.96%. When pH was 2, the killing rate of the PHMB disinfectant was 41.83% for *E. coli* ATCC 8099 and 100% for *S. aureus* ATCC 6538, *S. agalactiae* ATCC 12386, and *S. dysgalactiae* ATCC 35666 within 5 min.

### 2.4. Skin Disinfection Test

Under the condition of simulated skin disinfection, three contagious pathogens (*S. aureus*, *S. dysgalactiae*, and *S. agalactiae*) and one environmental pathogen (*E. coli*) were applied to the skin of rabbits to evaluate the bactericidal effect of PHMB disinfectant. The results of simulated skin disinfection on site are shown in Table 4. The PHMB teat disinfectant applied on the skin of rabbits with *E. coli* ATCC 8099, *S. aureus* ATCC 6538, *S. agalactiae* ATCC 12386 and *S. dysgalactiae* ATCC35666 achieved an average log_10_ reduction of greater than 4.

### 2.5. Teat Swabbing Procedure

After 30 s of PHMB immersion, the bacteria of cow breasts were counted prior to disinfection and 10 min and 12 h after disinfection, respectively. The mean log_10_ reduction in bacteria on the skin surface of 12 cows was 0.99 to 3.52 after applying the PHMB disinfectant for 10 min (Table 5). After 12 h, the PHMB disinfectant achieved an average log_10_ reduction in bacteria from 0.27 to 0.68 (compared with that prior to disinfection). In the PHMB solution group, the reattachment of bacteria to the breast can be observed. During the experiment, cows did not show any behavioral abnormality after applying the PHMB disinfectant and PHMB solution. No adverse reactions, such as redness and swelling, were found in the eyes of the cow udder. The cow udder felt warm and soft before and after the test, and the skin surface of the cow udder was smooth.

## 3. Discussion

Disinfectant bactericidal components used for the treatment and prevention of bacterial mastitis should have a wide antibacterial spectrum and are not prone to drug resistance [22]. The physicochemical effects of PHMB on the phospholipid membranes and DNA replication or repair mechanisms prevent or hinder the development of drug-resistant strains [23]. Reports showed that PHMB is the best antibacterial agent for long-term use by comparing the antibacterial effects of several antibacterial agents such as triclosan, octenidine, PHMB, PVP-iodine and chlorhexidine digluconate [24]. When the film-forming disinfectant is used for the breast skin of dairy cows, it can not only kill the pathogenic microorganisms but also form a protective film on the breast surface in time to prevent the external pathogenic microorganisms from attaching to the skin again or entering the breast through the breast tube [25,26]. In this study, the film-forming effects of four film-forming agents were compared, and PVA-1788 was finally selected as the film-forming agent. The optimum concentration ratio of the film-forming material, humectant and antifreeze in the PHMB disinfectant was further determined via an orthogonal test. After verification, the prepared PHMB disinfectant has good stability.

The bacteria that cause the most common forms of mastitis are considered contagious and environmental pathogens [27]. Contagious pathogens, such as *S. aureus*, *S. agalactiae* and *S. dysgalactiae*, can survive and grow in the mammary gland. Therefore, a high risk of infection spreads from the infected to non-infected areas and from one cow to another during milking [28]. Environmental pathogens thrive in the environment, especially in the presence of cow manure. The most important of this group is *E. coli*, a variety of strains with varying pathogenicity in animals and humans [29]. We selected *E. coli* ATCC 8099, *S. aureus* ATCC 6538, *S. agalactiae* ATCC 12386, and *S. dysgalactiae* ATCC 35666 as indicator bacteria in the in vitro and skin-simulated disinfection effect tests. The PHMB teat disinfectant achieved a 99.99% reduction rate against four indicator bacteria isolates at relatively low concentrations (dilution in 1: 4800). One research reported that low PHMB concentrations (4 mg/L) killed almost 100% intracellular *S. aureus* (EMRSA-15 and USA300), and that PHMB entered the keratinocytes, localized with EMRSA-15 and remained continuously over 5 h [30]. One study reported MIC_90_ values of PHMB ≥ 0.5 µg/mL against mastitis-causing *S. aureus* [31]. Another study evaluated the antimicrobial activity of PHMB against mastitis-causing *Prototheca* spp., and the MIC_90_ values of PHMB was 2 µg/mL, which was lower than sodium dichloroisocyanurate (1400 µg/mL) and sodium hypochlorite (2800 µg/mL) against *P. bovis* isolates [32]. 

Four types of indicator bacteria were inoculated on the skin of rabbits to evaluate the bactericidal effect of PHMB teat disinfectant. The PHMB teat disinfectant can efficiently eliminate bacteria attached to the skin (4 > mean log_10_ reduction). The simulated disinfection test of rabbit skin proved the effectiveness of disinfectant on pathogens in animals. Disinfectants with excellent germicidal efficacy can rapidly kill pathogenic bacteria on the breasts of dairy cows and prevent pathogenic microorganisms on the breasts from breeding [1]. In this study, a film-forming agent was added during the preparation of disinfectants to achieve sterilization and prevent the re-adhesion of bacteria. After 10 min of applying PHMB teat disinfectant to the cows, the mean log_10_ reduction in bacteria was above 0.99. After the disinfection was completed, the cows were allowed to move freely for 12 h, and the samples were resampled for bacterial count. The PHMB teat disinfectant achieved an average log_10_ reduction in bacteria from 0.27 to 0.68 (compared with that prior to disinfection). In the PHMB solution group, most of the bacterial colonies had a significant rebound in the number of bacteria. This finding was probably because the PHMB solution was immediately air-dried after the disinfectant was applied, and the cow’s breast skin was reinfected with bacteria after 12 h of free activity. 

## 4. Materials and Methods

### 4.1. Chemicals and Materials

PHMB hydrochloride was supplied by Macklin Biochemical Co., Ltd. (Shanghai, China). Polyvinyl alcohol-1788 (PVA-1788) was purchased from Yingjia Industrial Development Co., Ltd. (Shanghai, China). Propylene glycol and glycerol were purchased from Damao Chemical Reagent Factory (Tianjin, China). Tryptic soy broth, tryptic soy agar (TSA) and luria broth (LB) agar were purchased from Guangdong Huankai Microbial Technology Co., Ltd. (Guangzhou, China). Fetal bovine serum was obtained from Thermo Fisher Technology Co., Ltd. (Shanghai, China).

### 4.2. Bacterial Strains and Growth Conditions

The four standard strains (*S. agalactiae* ATCC 12386, *S. dysgalactiae* ATCC 35666, *S. aureus* ATCC 6538, and *E. coli* ATCC 8099) were purchased from the Chinese Veterinary Culture Collection Center. The culture medium of *S. agalactiae* ATCC 12386 and *S. dysgalactiae* ATCC 35666 was TSA with 5% fetal bovine serum. The culture medium of *S. aureus* ATCC 6538 and *E. coli* ATCC 8099 was LB agar.

### 4.3. Preparation of PHMB Teat Disinfectant

In this study, 0.3 g of PHMB was added to a beaker with a small amount of water and heated to 70 °C on a magnetic stirrer (2X15-3, Shanghai Sile Instrument Co., Ltd., Shanghai, China) until it dissolved. Then, 6 g of PVA-1788 was added and stirred at a constant speed of 70 °C for 3 h prior to cooling overnight. Propylene glycol and glycerol were then slowly added to the beaker to create a solution of 30% and 5%, respectively. The final volume was diluted to 100 mL with water and continuously stirred until it was fully mixed, resulting in the creation of a PHMB disinfectant. The preparation of six batches of PHMB teat disinfectant is repeated in accordance with the above method. The PHMB content in the disinfectant was determined via spectrophotometry (UV-1780, Shimazu, Kyoto, Japan) [33].

### 4.4. Stabilization Test

The stability of PHMB teat disinfectant was assessed using an accelerated test method in accordance with the guidelines [34]. The PHMB teat disinfectant was stored in a 37 °C incubator under dark conditions. The concentration of PHMB was measured using a spectrophotometer (UV-1780, Shimazu, Japan) at 0 days and 90 days. A decrease rate of PHMB was deemed acceptable if it was below 10%.

### 4.5. Quantitative Suspension Test

The effectiveness of PHMB teat disinfectant in inhibiting bacterial growth against four types of bacteria (*S. agalactiae*, *S. dysgalactiae*, *S. aureus*, and *E. coli*) known to cause bovine mastitis [28] was studied. The PHMB teat disinfectant was dissolved in hard water at different dilutions and was combined with a solution of 4% soybean lecithin, 3% Tween-20, 3.6% sodium thiosulfate, and 1.25% anhydrous sodium sulfite in double-distilled water to inactivate or neutralize the antiseptic solution. The disinfectant was combined with the bacteria and incubated at a constant temperature of 20 °C for four periods of time (5, 10, 15 and 30 min). The test bacteria suspension (2.5 mL) and disinfectant (2.5 mL) were added to a sterile test tube and immediately mixed. After each time point, a sample solution (0.5 mL) was added to a test tube containing neutralizer (4.5 mL) and mixed for 10 min. The resulting solution (0.5 mL) was then added to a nutrient broth tube (4.5 mL) and incubated at 37 °C for 24 h to evaluate bacterial growth visually. The quantitative tests were also carried out on suspensions. Neutralization was performed for 10 min and incubated in a medium at 37 °C for 24 h to count viable bacteria. Each experiment was repeated three times. 

### 4.6. Factors Affecting Disinfection Effect Test

The determination of the factors influencing the efficacy of PHMB teat disinfectant was conducted in accordance with the guidelines of disinfection technology standards [34]. The film-forming effect is evident after dilution because PHMB teat disinfectant has film-forming properties. Therefore, an undiluted disinfectant was used for the influencing factor test. The organic substance protection test was conducted at 20 °C using different concentrations (5%, 10%, 15%, and 20%) of bovine serum albumin for quantitative analysis. In addition, the efficacy of the PHMB disinfectant was tested at different temperatures (−20 °C, −10 °C, 0 °C, 10 °C, 20 °C, 30 °C, and 40 °C) and pH values (pH = 2, 4, 6, 7, 8, 10) to determine its germicidal properties. 

### 4.7. Skin Disinfection Test

Eight healthy female rabbits weighing 2.2 ± 0.3 kg were obtained from Guangdong Medical Laboratory Animal Center, and all the animal experiments were conducted under the supervision and guidance of the South China Agricultural University Laboratory Animal Centre. Based on references and proper modification [35], the rabbit’s back was prepared for bacterial inoculation by shaving the hairs on both sides and washing the area with 75% ethanol to remove bacteria. A bacterial suspension of 10 μL (1 × 10^6^–1 × 10^7^ CFU/mL) was evenly applied to the test area using a one-time inoculation ring. The PHMB teat disinfectant was then applied for 10 min and allowed to dry. After disinfection, a sterile cotton rod dipped in neutralizer was used for sampling. The cotton rod was mixed well in a 5 mL centrifuge tube containing neutralizer, and 100 μL was coated on medium and cultured at 37 °C for 24 h. A control group was also set up using distilled water and subjected to the same treatment. All animal procedures were approved by the Institutional Animal Care and Use Committee of South China Agricultural University (approval number: 2021C078), and the animals were treated with consideration for their welfare and in compliance with all local and national legal requirements. 

### 4.8. Teat Swabbing Procedure

This study involved 12 healthy adult female Holstein cows weighing approximately 700 kg, provided by Guangzhou Yanhai Dairy Technology Research Co., Ltd. (Guangzhou, China). The cows underwent routine feeding and were provided free access to drinking water during the two-week observation period prior to the experiment. Twelve dairy cows were selected, and the milk areas on the left and right sides of each cow were designated as the experimental and control areas, respectively. Prior to the experiment, bacteria were counted via sterile cotton swab sampling. The left breast of each cow was bathed with PHMB teat disinfectant, whereas the right breast was treated with a PHMB solution (3 g/L) for the control group. The cow’s teat was fully immersed in disinfectant for 15 s with a teat pre-dip foam [36]. At 10 min and 12 h after disinfection, a sterile cotton rod dipped in neutralizer was used to collect samples from three different positions on the outer side of the breast. The samples were mixed in a tube containing 5 mL of neutralizer, and 100 μL was cultured on nutrient agar medium at 37 °C for 24 h for bacterial count. All animal procedures were approved by the Institutional Animal Care and Use Committee of South China Agricultural University (approval number: 2021C078), and the animals were treated with consideration for their welfare and in compliance with all local and national legal requirements.

## 5. Conclusions

In this study, a type of film-forming PHMB teat disinfectant was prepared with PHMB as the main disinfection component and PVA-1788, propylene glycol, and propylene glycol as excipients. The PHMB teat disinfectant had a good killing effect on four types of indicator bacteria colonized in rabbit skin. It has a good effect on the elimination of bacteria in dairy cow teats and the prevention of bacteria recolonization after disinfection. Further evaluation is required to assess the impact of this disinfectant on the prevention and treatment of pathogenic bacteria responsible for mastitis in clinical settings. Additionally, it is necessary to conduct further research on the pharmacokinetic properties of PHMB in dairy cows in order to obtain safety data and reference for the establishment of a withdrawal period.

## Figures and Tables

**Table 1:** Determination of PHMB teat disinfectant content (*n* = 6).

Disinfectant	Normal Concentration (g/L)	Day 0 Concentration (mean ± SD, g/L)	Day 90 Concentration (Mean ± SD, g/L)	Degradation Rate ^a^ (%)
PHMB teat disinfectant	3.00	2.83 ± 0.11	2.76 ± 0.08	2.47

^a^ Degradation rate = (Day 0 concentration–Day 90 concentration)/Day 0 concentration × 100%.

**Table 2:** Mean reduction rate achieved by PHMB teat disinfectant for *E. coli* at each exposure time and dilution by quantitative suspension test.

Strain	Exposure Time (min)	Reduction Rate ^a^ (%)			
		1:3200	1:4000	1:4800	1:5600
*E. coli* (ATCC 8099)	5	100	100	99.99	<99.90
	10	100	100	99.99	<99.90
	15	100	100	100	<99.90
	30	100	100	100	<99.90

^a^ The experiments were conducted three times, and an average (mean) reduction rate was obtained for each test condition.

**Table 3:** Mean reduction rate achieved by PHMB teat disinfectant for *S. aureus*, *S. agalactiae*, and *S. dysgalactiae* at each exposure time and dilution by quantitative suspension test.

Strain	Exposure Time (min)	Reduction Rate ^a^ (%)			
		1:6400	1:8000	1:9600	1:11,000
*S. aureus* ATCC 6538	5	99.99	99.99	<99.90	<99.90
	10	100	99.99	<99.90	<99.90
	15	100	99.99	<99.90	<99.90
	30	100	99.99	<99.90	<99.90
*S. agalactiae* ATCC 12386	5	100	100	99.99	<99.90
	10	100	100	99.99	<99.90
	15	100	100	100	<99.90
	30	100	100	100	<99.90
*S. dysgalactiae* ATCC 35666	5	100	99.99	99.99	99.91
	10	100	100	99.99	<99.90
	15	100	100	100	<99.90
	30	100	100	100	<99.90

^a^ The experiments were conducted three times, and an average (mean) reduction rate was obtained for each test condition.

**Table 4:** Mean log_10_ reduction achieved by the PHMB teat disinfectant for four strains via artificial simulation test.

Organism	Mean log_10_ Reduction
*E. coli* ATCC 8099	5.29
*S. aureus* ATCC 6538	4.53
*S. agalactiae* ATCC 12386	4.38
*S. dysgalactiae* ATCC 35666	4.10

**Table 5:** Mean log_10_ reduction achieved by PHMB teat disinfectant and PHMB solution for bacteria on cow breasts.

Cow	PHMB Disinfection (Mean log_10_ Reduction)		PHMB Solution (Mean log_10_ Reduction)	
	10 min	12 h	10 min	12 h
1	1.40	0.43	1.88	−0.10
2	2.72	0.38	2.34	0.08
3	3.52	0.27	1.85	0.10
4	2.09	0.56	2.48	0.01
5	1.46	0.45	1.25	0.10
6	2.50	0.33	2.09	−0.16
7	1.56	0.56	3.34	−0.02
8	1.21	0.57	1.68	0.18
9	1.93	0.57	1.73	−0.07
10	0.99	0.66	2.36	0.09
11	1.39	0.68	1.25	0.17
12	1.63	0.50	1.67	−0.03
Mean ± SD	1.87 ± 0.73	0.50 ± 0.13	1.99 ± 0.58	0.03 ± 0.11

## Data Availability

No new data were created or analyzed in this study. Data are contained within the article.
